# Staging of Prostate Cancer Using Automatic Feature Selection, Sampling and Dempster-Shafer Fusion

**DOI:** 10.4137/cin.s819

**Published:** 2009-02-03

**Authors:** Sandeep Chandana, Henry Leung, Kiril Trpkov

**Affiliations:** 1 Department of Electrical and Computer Engineering, University of Calgary, ICT-402, 2500 University Drive NW, Calgary, Alberta, T2N 1N4 Canada; 2 Department of Pathology and Laboratory Medicine, Calgary Laboratory Services, Calgary, Alberta T2V 1P9 Canada

**Keywords:** prostate cancer, staging, classifier fusion

## Abstract

A novel technique of automatically selecting the best pairs of features and sampling techniques to predict the stage of prostate cancer is proposed in this study. The problem of class imbalance, which is prominent in most medical data sets is also addressed here. Three feature subsets obtained by the use of principal components analysis (PCA), genetic algorithm (GA) and rough sets (RS) based approaches were also used in the study. The performance of under-sampling, synthetic minority over-sampling technique (SMOTE) and a combination of the two were also investigated and the performance of the obtained models was compared. To combine the classifier outputs, we used the Dempster-Shafer (DS) theory, whereas the actual choice of combined models was made using a GA. We found that the best performance for the overall system resulted from the use of under sampled data combined with rough sets based features modeled as a support vector machine (SVM).

## 1. Introduction

Prostate cancer is the leading type of cancer in men with a 27% incidence. The Canadian Cancer Society estimates about 72,700 cancer deaths, of which 3500 are in men with prostate cancer.^1^ There are several different types of treatments for prostate cancer, although some of the treatment techniques can substitute each other. The choice of a treatment is dependant upon the stage of the disease, i.e. the extent of cancer spread and whether the cancer has spread beyond the prostate. This information results in staging the cancer. In essence, on the information gathered about the disease through biopsy and/or prostatectomy, staging categorizes a patient. Since different treatments result in variable outcomes, staging helps assess the risk of cancer progression and death based on the current extent of cancer, tumor characteristics, metastasis in the lymph nodes, and the spread of the disease to other parts of the body. Staging also helps in establishing a tradeoff between the risks of death due to cancer and death or medical complications due to treatment. This is particularly true for prostate cancer since a majority of men diagnosed with the disease are older adults, often suffering from multiple comorbidities.

Typically, medical doctors assess the clinical and the pathological data about the individual patient to assign a cancer stage and to choose the most appropriate treatment procedure. This is also known as clinical decision making and it is a complex process. Cancer staging performed by a doctor involves weighing multiple variables and processing information gathered by patient examination and by conducting various tests. However, this process may be subjective and therefore depends heavily on the doctor’s experience, skills and knowledge. Machine learning techniques can also be employed to learn and model the underlying theory when provided with relevant information and data. A variety of statistical, probabilistic and optimization tools under the umbrella of machine learning can be used to learn from past examples and a number of information systems have been developed to aid the clinical decision-making process.^2^

An automated cancer staging system was developed and is described in this paper. A classifier based framework was used to draw a distinction between two primary stages of the disease: organ-confined disease and extraprostatic disease. The classifier was modeled using past data from patients diagnosed with prostate cancer who are selected to undergo surgery (or prostatectomy). One of the key issues with such data sets is the class imbalance, resulting from the number of patients with organ-confined disease, which considerably exceeds the number of patients with extraprostatic disease. Data imbalances pose problems when less observed patterns are of higher relevance, because most of the machine learning techniques tend to generalize the patterns observed over the majority of data and ignore those observed over smaller portions of the data.^3^ Three approaches of dealing with the class imbalance problem were explored in this study. We also investigated the feature space reduction, because the data can potentially contain more information than required to perform the classification.

There are a number of machine learning techniques that can be used to develop a classifier, but it is well known that specific techniques are more suitable for certain domains. That is, for a particular problem, specific techniques have superior performance, while other techniques are only able to produce mediocre or acceptable results. Even within one problem domain, different techniques can differ in the efficiency for different (partial) ranges of the problem space. This leads to the conclusion that it is most appropriate when solving a problem, such as the one considered here, to primarily rely on actual data and information about the problem, rather than trying to generalize the performance of certain machine learning techniques in a generic manner.

Lately, a number of machine learning based tools have been developed for cancer diagnosis and prediction. And a majority of this work can be categorized into those which are heavily dependant upon expert domain knowledge or on extensive historic data. Garzotto et al.^4^ and Spurgeon et al.^5^ sequentially developed a decision tree approach, specifically CART (classification and regression tree) to classify patients with aggressive prostate cancer based on ultrasound and biopsy markers. Sensitivity and specificity were adopted as performance metrics in the study. Zlotta et al.^6^ developed a set of two artificial neural networks (ANN), where the first ANN predicts the pathological stage of the patient and the second ANN classifies patients in groups based on cancer stage and the model performance was measured using the Receiver Operating Characteristic (ROC) curve. Veltri et al.^7^ favorably compared an ANN model to logistic regression for prostate cancer prediction and staging. Percentage of correctly classified cases was adopted as the performance metric.

In this paper, we propose a novel system which selects the features automatically and couples appropriate techniques in order to maximize the system performance. Specifically, performance of three re-sampling techniques and three feature selection techniques were evaluated. A GA is used to search for optimal pairs of feature selection and sampling techniques; where optimality is based on the performance of the system. Once such pairs are identified, respective classifiers are developed and a DS method^8^ is used to combine the component classifier performances. The performance of such a system has been shown for two different types of classifiers; the k-nearest neighbor (KNN) method and the support vector machines (SVM). ROC curves^9^ are used as performance measures for the proposed cancer staging system. The next section describes the data used for this study and Section 3 provides justification for the use of ROC as a performance metric. Section 4 introduces the proposed method along with brief descriptions of the component classifiers and feature extraction and sampling methods. Section 5 details the classifier fusion and the obtained results. Section 6 provides details on the generic applicability of this method by validating it on a simulated prostate cancer dataset and on two publicly provided datasets of breast and lung cancer.

## 2. Study Population

Staging and analysis of prostate cancer may be regarded as a function of the information (predictors) gathered during the diagnosis of every patient. Data from a total of 1174 patients with prostate cancer positive biopsies matched with their radical prostatectomies were used for this study. All specimens were processed in one laboratory between 07/2000 and 04/2005 and were reported using standard synoptic reports. The biopsy and the prostatectomy data were collected from the information system (Cerner) of the Calgary Laboratory Services. Various biopsy predictors that were considered are: Patient Age, Primary Gleason Grade, Secondary Gleason Grade, Biopsy Gleason Score, Prostate Specific Antigen (PSA), PSA Density (PSAD), Digital Rectal Exam (DRE), Transrectal Ultrasound (TRUS), Gland Volume, Number of Positive Cores, Total percent of Cores Involvement, and Total Cancer Length in mm. Prostatectomy data included: Disease Stage (pTNM), Primary Gleason Grade, Secondary Gleason Grade, Prostatectomy Gleason Score, Tumor Volume, Seminal Vesicle Involvement, Surgical Resection Margin Status, and Pelvic Lymph Node Involvement. The prostate cancer dataset contained multiple stages for the disease, and thereby the stage data was converted into a binary format where extraprostatic disease represented by stages pT3 and pT4 was denoted by 1 and the organ-confined disease represented by stage pT2 was represented with a 0. Table 1 presents the statistical description of the data used in this study. Of the 1174 patient records, 1054 records were retained after removing the ones with missing variables; 934 patients in the organ-confined stage and a 120 with an extra prostatic extension.

## 3. Performance Metric

A classifier’s performance typically reflects how well it can discriminate between the objects belonging to different classes (two in this case). But the proposed system is expected to play a critical role, particularly for patients where treatment modality may have a substantial impact on the post-treatment prognosis and survival. Therefore, in terms of the classification performance, the cost of wrongly classifying a patient with extraprostatic disease is much higher than the cost of wrongly classifying one in the organ-confined stage. Conventional measures of performance therefore will not provide a relevant estimate of the classifier performance; therefore an ROC curve and the area under the curve (AUC) were used as performance indices in this study.

An ROC curve is obtained by plotting the true positive rate (TPR) against the false positive rate (FPR) for varying decision thresholds. As shown in Table 2, TPR (and FPR) is representative of the number of positive (negative) examples correctly (incorrectly) classified. They may be computed as follows,

$$\begin{array}{l} TPR=\frac{TP}{TP+FN};\\ FPR=\frac{FP}{FP+TN}; \end{array}$$

The decision threshold or boundary for binary classification refers to a threshold, below which the object is classified as negative and above which it is classified as positive. Such a threshold can be adjusted to trade off the cost of *TP* against the cost of *FP*, and each threshold setting provides a (*FP*, *TP*) pair. A series of such pairs produced by varying the decision threshold are used to plot the ROC curve. The ideal point on the ROC curve would be (0, 1) where all positive examples are classified correctly and no negative examples are misclassified as positive. (0, 0) is the point where all the examples are predicted as negative. (1, 1) corresponds to classifying all examples as positive.

One should note that, depending on the outcome of misclassification, ideal decision thresholds may vary. For example, if the cost of misclassifying a patient with extraprostatic disease is lower than misclassifying a patient with organ-confined disease, then a reciprocal ROC curve (to the one discussed above) will be preferred. But in this study, a classifier with an ROC tending towards the top-left of the graph indicates better performance than the ones with a lower ROC. In addition, ROC curves for different classifiers tend to intersect each other; in which case AUC is used as an alternate metric. AUC ranges between the interval [0, 1] and greater the value of AUC, better is the technique. The AUC of a classifier is equivalent to the probability that the classifier will rank a randomly chosen positive example higher than a randomly chosen negative example. AUC as a measure has been proved to be equivalent of the Wilcoxon test statistic^10^ and the Gini Index^11^ i.e. unlike a typical measure of classification accuracy, the AUC quantifies the likelihood that the underlying method will assign a higher probability of success to a patient having extraprostatic disease compared to a patient where the cancer is contained. Therefore such a measure will provide a true insight even in the case of imbalanced data. Another important advantage is that the respective ROC is invariant to monotone transformations of feature values,^12^ which renders flexibility in manipulating the feature set if necessary.

## 4. Proposed System and Methodologies

The proposed system consists of four major parts: feature extraction, data sampling, classification and classifier fusion. Feature extraction helps identify the most prominent features in the search space thereby reducing the required computational and interpretational effort. Data sampling provides a mechanism to eliminate the bias or imbalance that exists in the data by over-sampling the minority class or under- sampling the majority class or a selective combination of both. Feature extraction and sampling enable the implementation of a classifier in order to model the class disparity in the data. A GA is then used to identify compatible sets of features, sampling techniques and classifiers in order to maximize the performance of subsequent DS classifier fusion. The adopted methods are individually described in the following subsections.

### A. Feature selection

Features extracted or selected from the input data, can be categorized into continuous, discrete or projected features. Existing processes of selecting features can be classified as: those based on statistical information, those based on empirical information and those based on search in the sample space. Following this approach, three techniques associated with the major types of features have been adopted. Selection of RS based discretized features relies on empirical information about the system, PCA based transformed feature selection relies on the statistics of the data and GA based continuous feature selection relies on intelligent search through the sample space.

#### 1. RS features

Rough Set Theory^13^ adopts an equivalence relation such that two objects (*x*, *y*) form an indiscernible pair over the attribute *a*, only if *a*(*x*) *= a*(*y*); and assuming that (*x*, *y*) ∈ Ra, then Ra would be called the *a*-indiscernibility relation and denoted by the symbol INDa. Given that, a lower approximation set consists of all objects which can certainly be classified as elements of *X* over an indiscernibility relation *R*, i.e.

(1) $$\underline{R}\;X=\cup \;\{Y\in U|R:Y\subseteq X\}$$

The lower approximation set is also known as the Positive region i.e.

(2) $$POS_{R}(X)=\underline{R}\;X$$

The significance of a variable is then expressed as a function of the dependency (γ) of a variable in classifying the objects into the classes of *U* |*IND*(*D*). The dependency of decision variable *D* on independent variable *R* is given as:

(3) $$\gamma_{R}(D)=\frac{\left|POS_{R}(D)\right|}{\left|U\right|}$$

where |*U*| denotes the cardinality of set *U*, i.e. the number of elements contained in that set. The significance of a variable *a* is the increase of dependency between the independent variables and the decision variable after the addition of *a*, i.e.

(4) $$SGF(a,R,D)=Y_{R+\{a\}}(D)-Y_{R}(D)$$

Because dependency (defined by Equation 3) only considers the number of rules that cover various instances and not the number of instances that each of the rules represents, a parameterized average support heuristic method has been adopted to include both, the number of rules and the number of instances supporting each rule in computing the average support function (similar to the measure of dependency), given as:

(5) $$F_{R}(D)=|POS_{R}(D)|\times \frac{1}{n}\sum_{i=1}^{n}S(R,d_{i})$$

where *d_i_* is a possible value of decision variable *D*, and *S*( *R, d_i_* ) = max{|[*x*]*_IND_*_(_*_R_*_)_|:[*X*]*_IND_*_(_*_R_*_)_ ⊂ *POS_R_* (*D* = *d_i_*)} indicates the maximal size of the equivalence classes included in the positive region of {*D* = *d_i_*}i.e. the support of the most significant rule for the decision class {*D* = *d_i_*}. The significance of a variable (Equation 4) is redefined as:

(6) $$SGF(a,R,D)=F_{R+\{a\}}(D)-F_{R}(D)$$

#### 2. PCA features

PCA^14^ reduces the feature space by projecting the complete feature set onto fewer variables known as the principal components with the objective of maximizing the variance in a least squares sense. This produces uncorrelated components with minimal information loss. *X* denotes a {*n* × *p*} matrix for *n* instances of a system represented through a p-dimensional feature space. Applying PCA begins by first normalizing *X* into a feature set with zero mean and unit variance. PCA aims to transform this p-dimension into an m-dimensional feature space where *m* ≤ *p*, but typically the first few components represent the largest portion (∼90%) of the original information, therefore effectively using only the first m* (≪ *p*) components. The correlation matrix *S_X_* of *X* is given as:

(7) $$S_{X}=\frac{X^{T}X}{n-1}$$

If *Y* represents the {*n* × *m*} matrix for *n* instances of a system represented through a reduced m-dimensional feature space, the transformation is achieved by weighting the original features using m number of principal components. The m components are identified as the eigenvectors corresponding to the first m largest eigenvalues of the correlation matrix *S_X_*. The transformation of the feature space is therefore given as:

(8) $$Y=V_{m}\cdot X$$

where *V_m_* is a {*p* × *m*} matrix made up of the *m* eigenvectors. Singular value decomposition of *X* is performed as:

(9) $$X=U\cdot L\cdot V^{T}$$

where *U* is column-orthogonal matrix of size {*n* × *p*}, *L* is a square diagonal matrix of size {*p* × *p*} where the diagonal elements are square roots of the eigenvalues of the correlation matrix *S_X_* and *V* is also a square matrix of size {*p* × *p*} where the columns correspond to the eigenvectors of the correlation matrix. *V_m_* consists of the first *m* eigenvectors in *V*. The number of principal components or eigenvectors (*m*) is determined as per a set threshold.

#### 3. GA features

GA^15^ based search depends on a user defined fitness function; in this study, the product of the number of features and the average error in the predicted output class has been adopted as the fitness function. GA based search uses a chromosome representation of the solutions and a set of genetic manipulations in order to arrive at an optimum solution. First, a chromosome of the length equal to the total number of features in the input space is created. The value of the bit associated with a feature is set to 0 to indicate that the feature is not considered, whereas a bit value of 1 indicates that the feature will be considered. The search process begins with an initial generation where the population is generated randomly; all of the chromosomes in this generation are evaluated against the fitness function and the best chromosomes (representing better solutions) are chosen to propagate into the next generation. Through heuristic manipulation of the chromosome structures in every generation, it is ensured that the newer generations always have an average fitness higher than the previous generations. The search stops either when a fitness threshold is achieved, or when the search runs out of the threshold on the number of generations. In this study, the population size per generation was set to 25 and the limit on the generations used in the search was set to 1000. These numbers were selected after a short sequence of random trials. Mutation and crossover operators were utilized to generate off springs for the next generation, and a simple natural selection based on the current fitness values was used to identify potential parents.

### B. Data sampling

Two re-sampling methods are often used in order to overcome an imbalance; one is to under-sample the majority class to match the size of the minority class and the other is to over-sample the minority class to match the size of the majority class. Over-sampling and under-sampling techniques have been previously evaluated for imbalanced datasets,^16^ and a conclusion that both methods were effective was drawn. In one study,^17^ combined over-sampling of the minority class and under-sampling of the majority class was used, but the combination did not provide significant improvement in the performance. Over-sampling in these cases was done by duplicating the original examples from the minority class, which does not cause minority class decision boundary to spread into the majority class region, but instead creates decision regions similar to those existing for the minority class. This shortfall may be overcome using an approach called SMOTE, as proposed in one study.^18^

SMOTE is an over-sampling of the minority class by creating “synthetic” examples. SMOTE is actually an interpolation approach where the synthetic examples are created along the line segments joining the example under consideration and any/all of its *k* nearest neighbors in the minority class. The synthetic examples are created in the following manner:

1. For each minority class example, find its *k* nearest neighbors in the minority class.
2. Randomly choose m (*m* < *k*) examples from the *k* nearest neighbors depending upon the over-sampling amount. For instance, if the required over-sampling amount is 200%, then only 2 neighbors are randomly selected from the *k* nearest neighbors.
3. Calculate the differences between the minority class example under consideration and its m nearest neighbors, which are randomly chosen.
4. Multiply the differences by a random number between 0 and 1, and add the results to the minority class example under consideration to produce m synthetic examples for this minority class example.

In this study, we used three re-sampling techniques: 1) under-sampling 2) SMOTE and 3) combined under-sampling and SMOTE in the multi-classifier fusion diagnosis system.

### C. Classifiers

Classification, an operation of assigning an unknown sample to one of the output classes can typically be performed by either fitting a model around the independent variables or through averaging or majority voting. In order to exemplify the proposed system for both types, we used SVM, which identifies a nonlinear model of the input variables, and KNN, which is based on majority voting.

#### 1. Support vector machines

SVM^19^ is a supervised machine learning methodology used for classification and regression. Compared with the traditional statistical and neural network methods, SVM has the advantage to effectively avoid a local minimum due to its convexity property. On the other hand, SVM uses the kernel trick to apply linear classification techniques to nonlinear classification problems. When Gaussian kernels are used, the resulting SVM corresponds to a radial basis function (RBF) network with Gaussian radial basis functions. In comparison with traditional RBF network, SVM has the ability to automatically determine the model size by selecting the support vectors based on quadratic programming (QP) procedure. Hidden neurons correspond to the support vectors. The support vectors serve as the centers of basis function in the RBF network. For linear SVM, the decision function is given in a linear form as:

(10) f(x)=w·x+b

The decision value produced by SVM is not the estimate of posterior probability. Here, the binning technique is used to transform the output of the SVM into calibrated probability.^19^ The binning technique proceeds by first sorting the training examples according to their decision values, and then dividing the value range into *k* equal sized intervals or bins. Given an example *x*, place it in a bin according to its decision value. The conditional probability of *x* is estimated as:

(11) $$p(c(x)=1|x)\frac{m}{n}$$

where *n* is the number of training examples that fall within the bin, *m* is the number of positive examples among these *n* training examples.

#### 2. *K*-nearest neighbor

KNN^20^ is a statistical method for classifying objects based on their *k* nearest training examples in the feature space. KNN classifies a new example by first calculating the distances of the new example from all other examples in the training set, and then selects *k* nearest training examples. The class of the new example is the most frequent class label presented among the *k* nearest examples:

(12) $$c(x)=\omega_{m},\;\;\;k_{m}={\text{max}}_{i=1}^{s}k_{i}$$

where *s* is the number of classes, *k_i_* is the number of examples belonging to class ω*_i_* among the *k* nearest examples, ∑*k_i_* = *k; i =* 1… *s*. The output of KNN classifier is not probability. For the two-class problem, the conditional probability can be directly estimated as:

(13) $$p(c(x)=1|x)\frac{k_{1}}{k}$$

where *k*_1_ is the number of positive examples among the *k* nearest neighbors of *x* and SMOTE in the multi-classifier fusion diagnosis system.

### D. Classifier fusion

Suppose the universal set Θ = {*A*_1_… *A_m_*} is a set of all propositions under consideration and its power set 2Θ is formed by all possible subset of Θ, including the empty set Ø, a one-element subset {*A_i_*} is called a singleton and a subset {*A_k_*, *A*_l_} represents a proposition denoting the disjunction *A_k_* ∪ *A*_l_ (*k*, l ∈{1, 2 … *m*}). DS theory assigns a numerical value to each subset of the power set 2Θ using mass function or basic probability assignment *m*: 2Θ → [0, 1]. The mass function has the following properties:

(14) $$m(\emptyset )=0,\sum_{A\;\in \;2^{\Theta }}m(A)=$$

Subsets *A* ∈2^Θ^ that satisfy the condition *m*(*A*) > 0 are called the focal elements of mass function *m*. Since a subset *A* is the disjunction of all its elements. If the proposition *B* ⊆ *A* is true, then the proposition *A* is also true. Hence, given a subset *A*, the belief *bel* (*A*) is defined as the sum of all the masses of its subsets:

(15) $$bel(A)=\sum_{B\;\subseteq \;A}m(B)$$

The belief value *bel*(*A*) indicates the degree that evidence supports the proposition *A*. When two evidences exist, there will be two different mass functions *m*_1_ and *m*_2_. If these two evidences are independent of each other, then the two mass functions can be combined into a new mass function *m* using Dempster’s rule^21^:

(16) $$\begin{array}{l} m(A)=(m_{1}⊕m_{2})\;(A)\\ =\frac{1}{K}\sum_{A_{1}\;\cap \;A_{2}=A}m_{1}(A_{1})m_{2}(A_{2}) \end{array}$$

where

$$\begin{array}{l} K=\sum_{A_{1}\;\cap \;A_{2}=\emptyset }m_{1}(A_{1})m_{2}(A_{2})\\ =1-\sum_{A_{1}\;\cap \;A_{2}\neq \emptyset }m_{1}(A_{1})m_{2}(A_{2}) \end{array}$$

is a normalizing factor, which measures how much *m*_1_(*A*_1_) and *m_2_* (*A*_2_) are in conflict with each other. So *K* is also called the conflict measure. If *K* = 0, the combination of *m*_1_ and *m*_2_ does not exist, it means *m*_1_ and *m*_2_ are totally contradictory. When more than two evidences exist, the mass function can be combined by sequentially using the formula:

(17) $$m=m_{1}⊕m_{2}⊕\cdots ⊕m_{k}$$

#### Multiple classifier fusion

For the prostate cancer stage prediction problem, there are two exhaustive and mutually exclusive propositions *A_i_*. Proposition *A*_1_ denotes that input example *x* is negative, and proposition *A*_2_ denotes that input example *x* is positive. The power set 2Θ = {Ø, {*A*_1_}, {*A*_2_}, {*A*_1_, *A*_2_}}. The key step in the DS fusion process is to assign a basic probability assignment (BPA) to each subset of 2Θ. After calibrating, if the outputs of each of the classifiers as conditional probabilities *p* (*A_i_*|*x*), let *D_k_* (*x*) = [*d_k,_*_1_(*x*), *d_k_*_,2_(*x*)] denote the output of classifier *ck*, then *d_k_*_,1_(*x*) + *d_k_*_,2_(*x*) = 1; *k* = 1, 2. Given classifier *ck*, assign the BPA values of subset {*A*_1_} and {*A*_2_} as:

(18) $$\begin{array}{l} m_{k}(A_{1})=d_{k,1}\\ m_{k}(A_{2})=d_{k,2} \end{array}$$

where *m_k_*(*A_i_*) indicates the degree of belief that proposition *A_i_* is true, provided by classifier *ck*, *k* = 1, 2. Combining the evidence provided by the individual classifiers, the belief value of each proposition is given as:

(19) $$bel\;(A_{i})=m(A_{i})$$

where *m* is the combined mass function calculated by the sequential use equation (hello)

(20) $$\begin{array}{l} m=m_{1}⊕m_{2}⊕m_{3}\\ =(m_{1}⊕m_{2})⊕m_{3} \end{array}$$

The final decision is made according to the approach dealing with the imbalanced data. If re-sampling is used in the fused diagnosis system, the final decision is made by assigning the label of the class with the largest belief value:

(21) $$\begin{array}{l} c(x)=m;\;\;\;m=1,2\\ bel(A_{m})={\text{max}}_{i=1}^{2}bel(A_{i}) \end{array}$$

If the imbalance is dealt with by changing the decision threshold, the class of the example is determined by changing the threshold on belief value of the positive class:

(22) $$\{\begin{array}{ll} c(x)=1, & bel\;(A_{1})>T\\ c(x)=0, & else \end{array}$$

where *T* is the threshold.

## 5. Results

**Figure 1:**
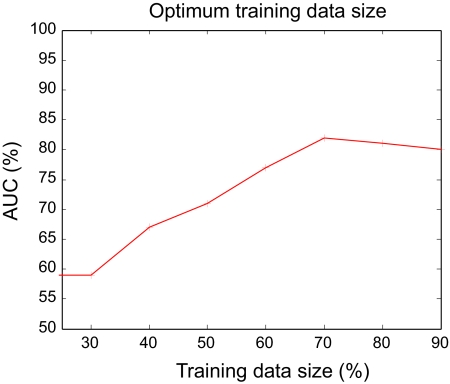
SVM performance for different training data sizes.

Experiments were done to assess the performance of all of the feature extractor-sampling-classifier pairs. The available data set was divided into two: one for building the models and the other to test the developed models. Appropriate ratio of the training and testing data sizes for SVM was identified by running different trials as shown in Figure 1. The best testing performance was observed when 70% of the total samples were used for training. The dip in the performance of the SVM beyond the training data size of 70% can be attributed to overfitting, when the trained model lost its ability to generalize, and was rather rigid to the training data. As the performance of the KNN depends on the number of neighbors considered in the output class allocation, the optimum number of neighbors was identified by running trials with different sizes, and 5 was the most optimal. Although higher number of neighbors may seem to have the ability to generalize, it is the separability of the data according to assigned classes that has the highest influence on the appropriate number of neighbors.

**Figure 3:**
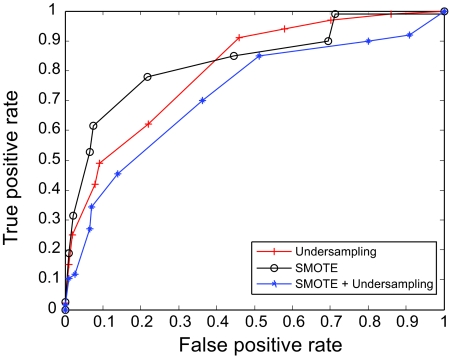
ROC curves of SVM and Rough Set Features.

**Figure 4:**
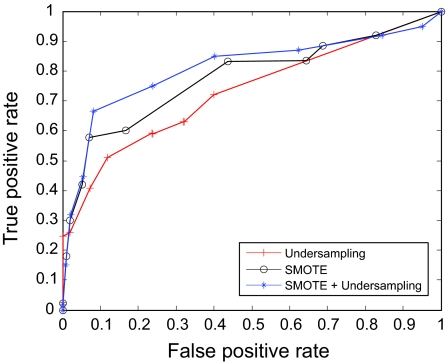
ROC curves of KNN and Rough Set Features.

**Figure 5:**
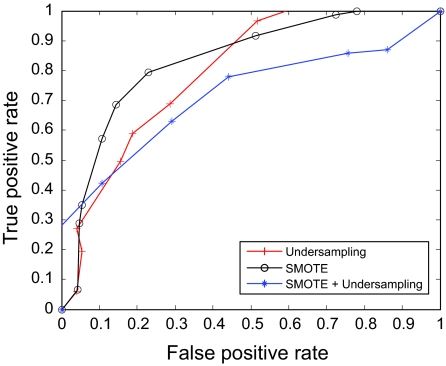
ROC curves of SVM and PCA Features.

**Figure 6:**
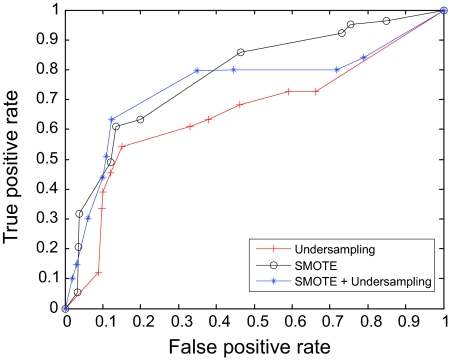
ROC curves of KNN and PCA Features.

**Figure 7:**
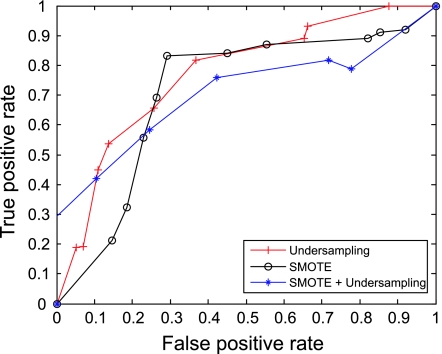
ROC curves of SVM and GA Features.

**Figure 8:**
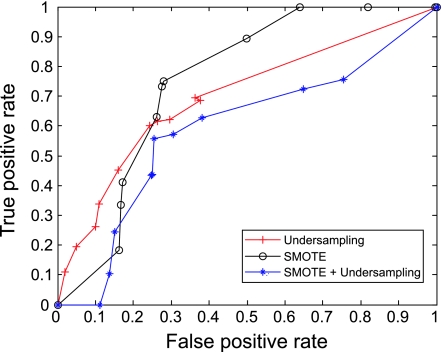
ROC curves of KNN and GA Features.

**Figure 9:**
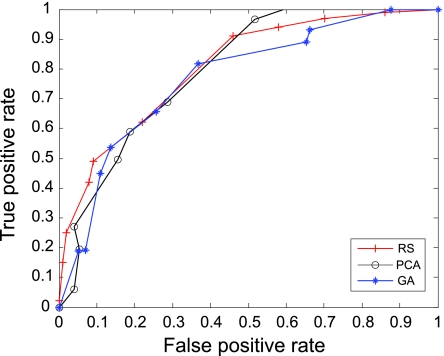
ROC curves of SVM and Under-sampling.

**Figure 10:**
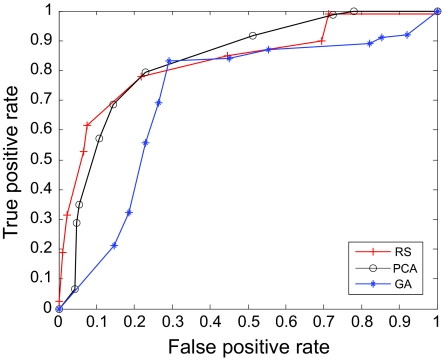
ROC curves of SVM and SMOTE.

**Figure 11:**
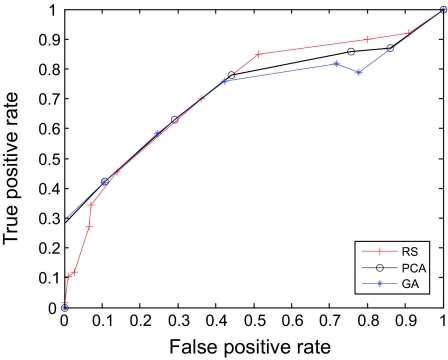
ROC curves of SVM and combined sampling.

**Figure 12:**
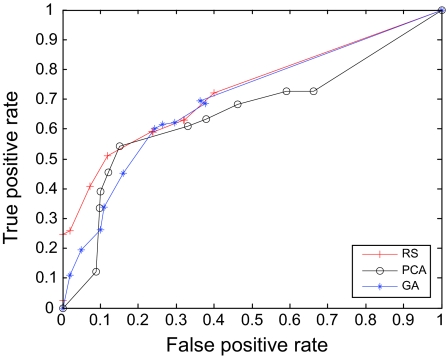
ROC curves of KNN and Under-sampling.

**Figure 13:**
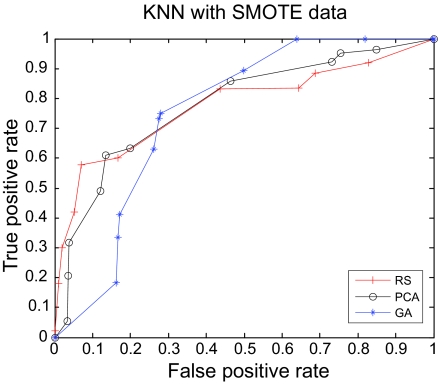
ROC curves of KNN and SMOTE.

**Figure 14:**
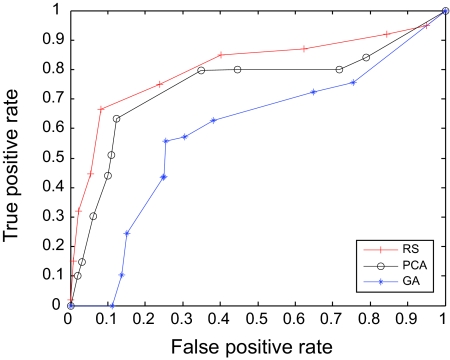
ROC curves of KNN and combined sampling.

The AUC curves were generated for all of the feature-sample-classifier sets by altering the decision threshold. When the outputs of the classifiers for all combinations were transformed into conditional probabilities, altering the decision threshold simply meant altering the respective probability threshold. By varying the probability threshold, the testing examples are re-labeled, giving out a series of (FP, TP) pairs. Each pair of (FP, TP) is a point on the ROC curve. For individual classifiers, ROC curves are created by changing the threshold on conditional probability *p* (*c*(*x*) = 1|*x*). ROC curve for the fused classifier is created by changing the threshold on belief value of the positive class. The outputs of SVM and KNN are then fused using DS fusion approach and ROC curves are plotted for the individual classifiers (Figures 3–14). AUC of the generated plots were tabled per classifier in Tables 3 and 4.

To show the effect of combining multiple sets of features and the effect of combining different types of sampling, DS fusion of the classifier outputs was performed over various combinations of sampling and feature selection methods. The following notation was adopted to refer to the classifiers developed in this study;

Notation: ‘C’ – ‘F’ – ‘S’.

where ‘C’ represents the type of classifier used, i.e. {SVM, KNN}; ‘F’ represents the type of feature selection tool used, i.e. {R (rough sets), P (principal component analysis), G (genetic algorithm)} and ‘S’ represents the type of data sampling adopted, i.e. {U (under sampling), S (SMOTE), US (under-sampling + SMOTE)}. Therefore SVM-P-S would imply the classifier based on SVM trained over PCA features obtained from SMOTE sampled data.

It is evident in Table 3 that the SVM trained over RST based features had a superior performance. Similarly, under-sampling combined with SMOTE trained SVM had the best performance among the types of sampling. DS fusion of SVM-R-[U, S, US] has 86.1% under favorable AUC. KNN performed poorly as a classifier over all sampling and features. Although much lower than average SVM, the combination of KNN-R-S has the highest favorable AUC at 80.9%, as illustrated in Table 4. In general, DS fusion improved the performance over any single model. We also note that under sampling proved a more efficient method of re-sampling the data than generating synthetic samples. As it has been observed previously,^18^ generating synthetic samples can cause the decision boundary to spread, resulting in a poor performance. Despite the fact that fewer samples make parameter estimation difficult in SVM and neural networks, under sampling of the data should be preferred, since performance degradation is higher when using synthetic sampling than using SVM trained over smaller data sets.

### B. Comparison between different methods

Methodology proposed in this study relies on the use of GA to identify the most optimal set of classifiers for fusion, where fitness is defined as the overall fused performance. A total of 18 models (9 each for SVM and KNN with variations in the sampling method and features) were developed, and GA was used to choose the best set of fusion classifiers. Once an *x* set of classifiers for best fused performance were identified, the AUC (of ROC for changing thresholds) was determined for the test samples alone. The results for the overall performance from all model combinations obtained by fusing 2, 3, 4 and 5 models are shown in Table 5 (A) with the highest classification accuracy at 90.1% and a respective ROC AUC of 0.8640. Clearly, the number of fused models does not have a considerable impact on the performance, which is the reason why the trials were stopped after the fusion of 5 models. A combination of the outputs of 4 models has the best overall performance on the testing data. In this study, under sampling was observed to be the most efficient for data sampling. KNN (with average performance for all models much lower than SVM) seems to have contributed equally in the best overall performance. As observed from Tables 3 and 4, overall efficiency of rough sets based features was highest of all subsets. Rough sets based discretized features increase the distance between different output classes, and thus tend to impact the overall classification performance in a favorable manner.

In order to contrast the performance of the proposed method with existing techniques, a C4.5 decision tree and a two-layer neural network have been additionally developed using the same data. C4.5, similar to the likes of expert systems and nomograms, is a simple and transparent technique which can also be regarded as means of knowledge representation. ANN, similar to the likes of SVM and nonlinear regression, on the other hand is a complex nonlinear model. These two models have been chosen to represent the majority of present day classifiers. As can be observed from Table 5 (B), the proposed method fairs very well compared to the two other. Moreover, the performance of a 2-model combination (AUC: 0.8617, Accuracy: 89.4%) is better than that of the DS (C4.5 + ANN) method (AUC: 0.8580, Accuracy: 88.5%).

## 6. Validation with Other Datasets

A number of such techniques have been reported in the literature and these techniques perform very well for individual datasets that the techniques have been built for. Data used in the previous section is a large, unbiased and consecutive patient cohort originating from one institution. Therefore it should be comparable to other current patient data obtained from patients who undergo prostatectomy in the larger North American centers. Although currently we have no access to data sets from different hospitals, we present the results of applying the proposed method to three different cancer datasets to testify that the proposed method is generic in applicability and that it outperforms other existing techniques, Firstly, we considered a simulated prostate cancer (SimPCa) dataset. 1000 patient records were synthetically generated using a combination of the under-sampling and SMOTE methods. It was ensured that the overall statistics of the data remained consistent with the dataset reported in Table 1. In order to facilitate comparison, the same classifiers, feature extractors and sampling techniques were adopted. ROC curves were generated and the respective AUC given in Tables 6–8. The performance of the classifier is very similar to the results in Section 5. SVM trained over RST identified features had the best performance and KNN remained a weak classifier for this dataset. Consistent with the other results, GA optimization identifies the same four model fusion.

The other two datasets considered for this purpose have been adopted from the University of California-Irvine data repository. Using the two public datasets we demonstrate that the proposed method compares favorably to the existing techniques. The Wisconsin Breast Cancer (BCa) data^22^ consists of 683 patient records, each described using a set of 9 features. The objective was to accurately distinguish patients suffering from benign and malignant breast cancer. The Hong-Yang Lung Cancer (LCa) data^23^ consists of 32 patient records, each described with a set of 56 features. The objective was to classify the patients into groups of three types of lung cancer. Tables 9–10 present the performance of SVM and KNN as classifiers over all sampling and feature extraction combinations for the breast cancer data and Tables 13–14 for the lung cancer data. Tables 11 and 15 depict the performance of the GA optimized classifier fusion.

It can be observed from the above Table 9 that SVM outruns KNN as a classifier and the best SVM classifier was built using GA for feature extraction and under-sampling as a remedy for data imbalance. Unlike in the prostate cancer data, GA optimization identified a three model fusion with the same AUC as for higher combinations. The best overall classification accuracy was 99.4%. Table 12 summarizes the performance of different methods in the literature for the same data. Not all the works listed in Table 12 use ROC as a metric; therefore classification accuracy has been used to draw a comparison between all of them. Quinlan^24^ and Pena-Reyes and Sipper^25^ applied C4.5 decision tree and fuzzy-GA methods. Although these methods are relatively simpler and yield a transparent and user-friendly model, the overall accuracy of the methods is compromised. On the other hand, Goodman et al.^26^ Ubeyli,^27^ Polat and Gunes^28^ and Akay^29^ applied thoroughly non-linear techniques. But it is evident that the proposed method, although marginal compared to Akay,^29^ has a superior performance than the other techniques.

For the lung cancer data, KNN and SVM have a similar performance. The combination of under-sampling and SMOTE with PCA feature extraction had the best performance for SVM as a standalone classifier, whereas the combination of SMOTE and PCA feature extraction had the best performance for a single KNN based classifier. GA optimization yielded a four model fusion as shown in Table 15 with an overall classification accuracy of 97.5%. Table 16 compares the performance of the proposed method to two other methods from the existing literature. The proposed method has a much better performance when compared to that of Aeberhard’s^30^ RDA model and the neuro-fuzzy model adaptation used by Luukka.^31^ Although Luukka^31^ reports a 99.99% accuracy for a larger training-testing ratio, a performance of 65.48% is given for a 70:30 ratio, which is the same as used for this work. Therefore, it is concluded that the proposed GA based optimization of multiple model fusion is generically applicable across a wide range of data and in addition ensures better performance than most existing techniques.

## 7. Conclusions

A number of classifier models based on KNN and SVM have been developed to test the automatic prediction of cancer stage using different features and data samples. We propose a novel approach of using a GA to select the best models and to combine their outputs using the DS theory. Owing to DS fusion and GA optimization, the overall performance improved in all tested models. In particular, three sampling and three feature selection methods have been employed in this study. Under-sampling and rough sets based features were identified to be most useful in improving overall performance of the system.

## Figures and Tables

**Figure 2:**
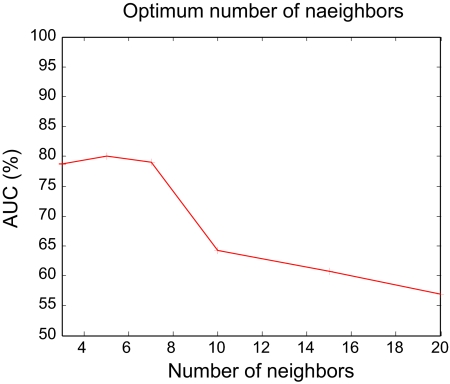
KNN performance for different number of neighbors.

**Table 1:** Patient characteristics.

Clinical parameters	Patients with Organ confined PCa (n = 934)	Patients with Extraprostatic extension (n = 120)	All patients (n = 1054)
Age (years): median (range)	59.9 (36.2–77.4)	63.4 (42.7–74.2)	60.4 (36.2–77.4)
Age categories (years):			
≤50 (%)	103 (11)	5 (4.2)	108 (10.2)
>50–60 (%)	368 (39.4)	34 (28.3)	402 (38.1)
>60–70 (%)	404 (43.3)	67 (55.8)	471 (44.7)
>70–80 (%)	59 (6.3)	14 (11.7)	73 (6.9)
PSA (ng/ml): median (range)	5.7 (0.29–55)	6.9 (1.8–80)	5.8 (0.29–80)
PSA categories (ng/ml):			
≤4 (%)	169 (18.1)	12 (10.0)	181 (17.2)
>4–10 (%)	662 (70.9)	73 (60.8)	735 (69.7)
>10–20 (%)	98 (10.5)	27 (22.5)	125 (11.9)
>20–50 (%)	3 (0.3)	6 (5.0)	9 (0.9)
>50–100 (%)	2 (0.2)	2 (1.7)	4 (0.4)
Prostate Gland Volume (cc): median (range)	35.6 (7–193.2)	32.1 (10.02–176.6)	35.15 (7–193.2)
Prostate Gland Volume categories (cc):			
≤25 (%)	188 (20.1)	33 (27.5)	221 (21.0)
>25–50 (%)	503 (53.9)	61 (50.8)	564 (53.5)
>50–100 (%)	243 (26.0)	26 (21.7)	269 (25.5)
PSA Density: median (range)	0.15 (0.01–2.1)	0.22 (0.03–2.5)	0.16 (0.01–2.5)
PSA Density categories:			
≤0.10 (%)	231 (24.7)	18 (15.0)	249 (23.6)
>0.10–0.25 (%)	535 (57.3)	49 (40.8)	584 (55.4)
>0.25–0.50 (%)	139 (14.9)	38 (31.7)	177 (16.8)
>0.50–1.0 (%)	25 (2.7)	11 (9.2)	36 (3.4)
>1 (%)	4 (0.4)	4 (3.3)	8 (0.8)
PCa Length (mm): Median (range)	5.25 (0–117)	19 (0.15–102.75)	6 (0–117)
PCa Length Categories:			
≤10 (%)	615 (65.8)	41 (34.2)	656 (62.2)
>10–20 (%)	174 (18.6)	25 (20.8)	199 (18.9)
>20–40 (%)	107 (11.5)	37 (30.8)	144 (13.7)
>40–60 (%)	31 (3.3)	12 (10.0)	43 (4.1)
>60 (%)	7 (0.7)	5 (4.2)	12 (1.1)
No. of cancer-positive cores: median (range)	2 (1–10)	4 (1–10)	2 (1–10)
No. of cancer-positive cores categories:			
≤4 (%)	767 (82.1)	74 (61.7)	841 (79.8)
>4–6 (%)	109 (11.7)	27 (22.5)	136 (12.9)
>6 (%)	58 (6.2)	19 (15.8)	77 (7.3)
Percent total core involvement on biopsy: median (range)	3.5 (0–78)	12.5 (0.1–68.5)	4 (0–78)
Percent core involvement categories:			
≤3 (%)	441 (47.2)	20 (16.7)	461 (43.7)
>3–10 (%)	280 (30.0)	30 (25.0)	310 (29.4)
>10–20 (%)	140 (15.0)	42 (35.0)	182 (17.3)
>20 (%)	73 (7.8)	28 (23.3)	101 (9.6)
Biopsy Gleason Score: median (range)	6 (4–9)	7 (6–9)	6 (4–9)
Biopsy Gleason Score categories:			
≤6 (%)	683 (73.1)	39 (32.5)	722 (68.5)
7–8 (%)	246 (26.3)	77 (64.2)	323 (30.6)
>8 (%)	5 (0.5)	4 (3.3)	9 (0.9)

**Abbreviations:** PSA, prostate specific antigen; PCa, prostate cancer.

**Table 2:** ROC confusion matrix.

		Predicted class	
		Positive	Negative
Actual class	Positive	TP	FN
	Negative	FP	TN

**Abbreviations:** TP, true positive; FN, false negative; FP, false positives; TN, true negatives.

**Table 3:** AUC values with SVM for PCa.

	Under	Smote	UnderSmote	DS
RST	0.8409	0.7223	0.8326	0.8611
PCA	0.8075	0.7439	0.8334	0.8392
GA	0.7704	0.7425	0.7112	0.7597
DS	0.8313	0.7461	0.8420	

**Table 4:** AUC values with KNN for PCa.

	Under	Smote	UnderSmote	DS
RST	0.7295	0.8088	0.7764	0.8065
PCA	0.6543	0.7450	0.7787	0.7891
GA	0.7383	0.7454	0.7560	0.7926
DS	0.7484	0.8001	0.7798	

**Table 5:** Performance of GA optimized fusion for PCa.

	2-models	3-models	4-models	5-models
Models	SVM-R-U	SVM-R-U	SVM-R-U	SVM-R-U
	SVM-R-US	SVM-P-US	SVM-P-U	KNN-R-S
		SVM-R-US	KNN-R-S	SVM-P-US
			KNN-G-U	SVM-R-US
				KNN-G-U
AUC	0.8617	0.8626	0.8640	0.8631
Accuracy	89.4%	89.7%	90.1%	89.8%
**A**. Comparison between different model combinations.				
	**Proposed**	**C4.5**	**ANN**	**DS (C4.5 + ANN)**

AUC	0.8640	0.8049	0.8359	0.8580
Accuracy	90.1%	83.0%	86.0%	88.5%

**Table 6:** AUC values with SVM for SimPCa.

	Under	Smote	UnderSmote	DS
RST	0.8376	0.7340	0.8312	0.8535
PCA	0.8076	0.7195	0.8304	0.8310
GA	0.7763	0.7707	0.7738	0.7790
DS	0.8403	0.7716	0.8336	

**Table 7:** AUC values with KNN for SimPCa.

	Under	Smote	UnderSmote	DS
RST	0.7290	0.8166	0.7705	0.8166
PCA	0.7130	0.7576	0.7701	0.7710
GA	0.8334	0.7358	0.7660	0.8346
DS	0.8360	0.8202	0.7740	

**Table 8:** Performance of GA optimized fusion for SimPCa.

	2-models	3-models	4-models	5-models
Models	SVM-R-U	SVM-R-U	SVM-R-U	SVM-R-U
	SVM-R-US	SVM-P-US	SVM-P-U	KNN-R-S
		SVM-R-US	KNN-R-S	SVM-P-US
			KNN-G-U	SVM-R-US
				KNN-G-U
AUC	0.8596	0.8620	0.8632	0.8610
Accuracy	88.8%	89.4%	89.8%	89.3%

**Table 9:** AUC values with SVM as the classifier for BCa.

	Under	Smote	UnderSmote	DS
RST	0.8917	0.9301	0.9680	0.9691
PCA	0.9342	0.9385	0.9360	0.9429
GA	0.9965	0.9737	0.9920	0.9965
DS	0.9965	0.9753	0.9921	

**Table 10:** AUC values with KNN as the classifier for BCa.

	Under	Smote	UnderSmote	DS
RST	0.9202	0.9243	0.9236	0.9270
PCA	0.9233	0.9230	0.9240	0.9240
GA	0.9289	0.9276	0.9318	0.9333
DS	0.9296	0.9290	0.9330	

**Table 11:** Performance of the GA optimized fusion for BCa.

	2-models	3-models	4-models	5-models
Models	SVM-G-U	SVM-G-U	SVM-G-U	SVM-G-U
	SVM-G-US	SVM-G-US	SVM-G-US	SVM-G-US
		KNN-G-US	KNN-R-US	KNN-R-US
			KNN-G-US	KNN-G-US
				SVM-G-S
AUC	0.9965	0.9994	0.9998	0.9998
Accuracy	99.1%	99.4%	99.4%	99.4%

**Table 12:** Classification accuracy for BCa.

Work (Year)	Method	Classification accuracy %
Quinlan^24^	C4.5	94.74
Pena-Reyes and Sipper^25^	Fuzzy-GA	97.36
Goodman et al.^26^	LVQ	96.80
Ubeyli^27^	EM-NN	98.85
Polat and Gunes^28^	LS-SVM	98.53
Akay^29^	SVM	99.02
Proposed method	GA-Fusion	99.40

**Abbreviations:** LVQ, linear vector quantization; EM, expectation maximization, LS, least squares.

**Table 13:** AUC values with SVM as the classifier for LCa.

	Under	Smote	UnderSmote	DS
RST	0.9265	0.9310	0.9305	0.9356
PCA	0.9000	0.9184	0.9376	0.9380
GA	0.9366	0.9298	0.9275	0.9349
DS	0.9366	0.9327	0.9401	

**Table 14:** AUC values with KNN as the classifier for LCa.

	Under	Smote	UnderSmote	DS
RST	0.9297	0.9140	0.9139	0.9321
PCA	0.9308	0.9384	0.9381	0.9396
GA	0.9350	0.9367	0.9330	0.9371
DS	0.9385	0.9390	0.9387	

**Table 15:** Performance of the GA optimized fusion for LCa.

	2-models	3-models	4-models	5-models
Models	KNN-P-S	KNN-P-S	KNN-P-S	KNN-P-S
	SVM-P-US	SVM-P-US	SVM-P-US	SVM-P-US
		KNN-P-US	KNN-P-US	KNN-P-US
			KNN-G-S	KNN-G-S
				SVM-G-U
AUC	0.9410	0.9427	0.9451	0.9451
Accuracy	96.6%	97%	97.5%	97.5%

**Table 16:** Classification Accuracy for LCa.

Work (Year)	Method	Classification accuracy %
Aeberhard et al.^30^	RDA	62.5
Luukka^31^	Yu-ANFIS	65.48
Proposed method	GA-Fusion	97.5

**Abbreviations:** RDA, regularized discriminant analysis; Yu, ANFIS- Yu norm ANFIS.
